# Transformational Leadership and Nurses’ Adaptive Performance: The Roles of Psychological Capital and Workplace Spirituality

**DOI:** 10.1155/jonm/8309473

**Published:** 2026-05-03

**Authors:** Anas Mahmoud Salem Abukhalifa, Mohammed Aboramadan

**Affiliations:** ^1^ Faculty of Business, Economics and Social Development, Universiti Malaysia Terengganu, Terengganu, Malaysia, umt.edu.my; ^2^ School of Economics, Administration, and Public Policy, Doha Institute for Graduate Studies, Doha, Qatar

**Keywords:** adaptive performance, nurses, psychological capital, transformational leadership, workplace spirituality

## Abstract

**Aim:**

This study examines the mechanisms through which transformational leadership enhances nurses’ adaptive performance within a conflict‐affected environment characterized by chronic uncertainty, escalating demands, and severe resource constraints. It introduces a novel‐mediated moderation model in which psychological capital (PsyCap) serves as a mediator and workplace spirituality (WPS) functions as a moderator.

**Background:**

Adaptive performance is vital in contemporary healthcare, yet its antecedents remain underexplored. While transformational leadership is recognized as a driver of employees’ psychological resources, little is known about how these resources translate into adaptive outcomes in nursing. This article represents one of the first empirical studies in nursing and management research to simultaneously test the direct effect of transformational leadership on adaptive performance, the mediating role of PsyCap, and the moderating role of WPS. By positioning WPS as a boundary condition, it demonstrates how spiritual values and meaning at work strengthen the influence of PsyCap on adaptability, marking a theoretical insight with practical significance.

**Methods:**

This study adopted a quantitative cross‐sectional design and surveyed registered nurses providing direct patient care in five major public hospitals across the Gaza Strip, Palestine. Data were collected via an online questionnaire using purposive sampling, and structural equation modeling was employed to test a moderated mediation model.

**Results:**

The results indicate that transformational leadership is positively associated with nurses’ adaptive performance and PsyCap. PsyCap partially mediates the relationship between transformational leadership and adaptive performance. In addition, WPS strengthens the positive association between PsyCap and adaptive performance, supporting the proposed moderated mediation model.

**Conclusions:**

Adaptive performance in healthcare can be enhanced when leadership, psychological resources, and spirituality are aligned.

**Implications for Nursing Management:**

Nursing leaders should deliberately enact transformational behaviors, systematically cultivate PsyCap, and nurture WPS to build resilient, adaptive nursing teams capable of thriving amid turbulence.

## 1. Introduction

Healthcare systems have become increasingly vulnerable to a confluence of complex disruptions, including global health emergencies such as the COVID‐19 pandemic, escalating geopolitical conflict, and persistent economic volatility. These multifaceted challenges have exposed critical structural deficiencies and intensified the operational demands placed on nursing professionals [1]. As primary agents in frontline care, nurses must navigate high‐stakes clinical environments, exercise sound judgment under time constraints, and manage the psychological burdens inherent in life‐threatening contexts [2]. Their ability to maintain clinical effectiveness, uphold care standards, and adapt to rapidly evolving conditions constitutes a vital pillar of organizational resilience and sustained healthcare delivery [3]. This challenge is particularly acute in conflict‐affected settings such as the Gaza Strip, where nurses deliver essential medical care amid warfare, critical shortages, and ongoing threats to personal safety [4]. In such environments, traditional models of job performance are insufficient to capture the breadth of capabilities required for effective functioning [5].

As a result, the construct of adaptive performance has garnered increasing scholarly attention, as it reflects the behavioral and psychological capacities essential for maintaining effectiveness under volatile and high‐stress conditions [6, 7]. Defined as the ability to modify behavior, assimilate new knowledge, and remain psychologically flexible, adaptive performance encompasses emotional regulation, cognitive agility, and innovative problem solving [8, 9]. It also involves stress management, continuous learning, and effective interpersonal functioning [5]. Despite its importance, empirical understanding of how adaptive performance is cultivated and maintained in conflict‐affected healthcare environments remains limited, particularly regarding its antecedents and mechanisms.

Given the psychological and operational challenges in high‐risk healthcare settings, transformational leadership has emerged as a critical driver of nurses’ positive attitudes and adaptive behaviors that underpin high‐quality patient care [10, 11]. Transformational leadership has been conceptualized through several influential scholarly traditions. Burns [12] first described it as a relational process in which leaders engage followers by addressing both existing and higher‐order needs, thereby elevating motivation and moral purpose. Building on this, Bass [13] framed transformational leadership as a style that reshapes followers’ values and beliefs, motivating them to exceed expectations. Bass and Riggio [14] further refined this perspective, highlighting a dynamic influence process through which leaders articulate a shared vision, stimulate followers intellectually, and provide individualized support to develop their capabilities. This approach is operationalized through four core behavioral dimensions: idealized influence, inspirational motivation, intellectual stimulation, and individualized consideration. Carless et al. [15] reinforced this framework by integrating these behaviors into a coherent and parsimonious model. Building on this conceptual foundation, transformational leadership is further theorized to produce superior outcomes relative to alternative approaches by activating followers’ intrinsic motivation, strengthening identification with collective goals, and facilitating adaptive responses under conditions of heightened occupational stress and strain [16]. Leaders exhibiting these behaviors are particularly influential in contexts characterized by instability, crisis, and environmental turbulence, where conventional managerial approaches are insufficient to mobilize adaptive behavior [14, 17, 18].

From the lens of Conservation of Resources (COR) theory [19], transformational leadership serves as a contextual resource that buffers nurses against the psychological strain of high‐stakes environments. COR theory posits that individuals strive to acquire and retain valued resources, particularly under threat. Resource loss without replenishment leads to elevated stress and diminished functioning [19]. Transformational leaders disrupt this cycle by reinforcing resource availability, instilling confidence, offering emotional support, and structuring meaningful work that restores psychological resilience [20]. They enhance role clarity, foster adaptive cognition, and promote a sense of control, facilitating both the preservation and generation of internal resources [21]. This leadership approach also enables nurses to reinterpret adversity as an opportunity for growth, thus promoting endurance and sustained clinical performance [22]. Although leadership has been linked to adaptability in various organizational settings (e.g., [6, 23]), limited evidence exists on how transformational leadership shapes adaptive performance specifically in healthcare sector.

To address this gap, we draw on COR theory [19] and introduce psychological capital (PsyCap) as a critical mediating mechanism linking transformational leadership to adaptive outcomes. PsyCap encompasses four interconnected psychological capacities (i.e., self‐efficacy, hope, resilience, and optimism) that enable individuals to navigate adversity, maintain goal‐directed focus, and respond effectively to dynamic demands [24]. Among nurses, PsyCap enhances job‐related attitudes and behaviors by fostering psychological readiness and promoting engagement in demanding work environments [25, 26]. PsyCap further plays a crucial role in promoting positive emotions, increasing constructive experiences, and expanding social relationships which foster employees’ well‐being [27]. Within COR theory, transformational leadership facilitates the development of personal resources by fostering a supportive climate. Leaders who offer constructive feedback, emotional support, and goal clarity help create the conditions necessary for resource gain [28]. PsyCap, in turn, enables nurses to regulate emotions, sustain motivation, and adapt to strain and complexity [7]. Its components collectively foster cognitive flexibility and behavioral agility that are critical for adaptive performance [29]. PsyCap also mitigates psychological depletion and initiates resource gain spirals, allowing sustained functioning under pressure [27]. Thus, while transformational leadership establishes the external conditions for resource acquisition, PsyCap enables individuals to internalize and mobilize those resources, thereby enhancing their adaptive performance. Despite its theoretical significance, the mediating role of PsyCap in this pathway remains insufficiently examined. To date, no empirical research has explicitly tested this mechanism among nurses in the global healthcare sector.

Given that PsyCap facilitates adaptive performance [30], its effectiveness might be contingent upon contextual factors that enable or constrain its activation. Drawing again on COR theory, we propose workplace spirituality (WPS) as a moderator influencing the PsyCap–adaptive performance relationship. COR theory suggests that resource gains are maximized in environments promoting psychological safety and value congruence [19]. WPS aligns with these conditions by fostering meaning, purpose, and a sense of connection, potentially amplifying the utility of internal resources [31, 32]. WPS refers to an organizational value framework that enables transcendence through work, creating feelings of wholeness and fulfillment [33]. It supports self‐realization beyond the material domain [34], particularly vital in emotionally taxing healthcare settings [35]. Besides, spiritual grounding can deepen the meaning of caregiving and sustain resilience [36]. Building on COR theory, we contend that WPS enhances the conversion of PsyCap into adaptive behavior by reinforcing value alignment, supporting emotional regulation, and promoting coherence. PsyCap provides the internal capacity to face adversity, while WPS offers contextual support for its activation. WPS strengthens this conversion by buffering against depletion and reducing compassion fatigue [37]. It nurtures hope, engagement, and flexibility, improving the ability to respond constructively to change [38]. Nevertheless, the moderating role of WPS remains underexplored in healthcare and organizational research.

**FIGURE 1:**
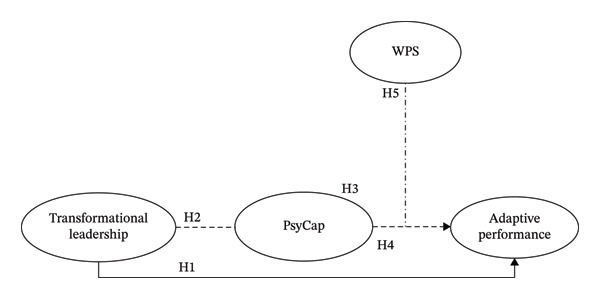
Research model. Note: PsyCap—psychological capital, WPS—workplace spirituality.

Overall, our study addresses these gaps by proposing a moderated mediation model wherein transformational leadership enhances adaptive performance through the development of PsyCap, with this indirect effect contingent on levels of WPS. As shown in Figure 1 and based on this framework, we propose the following five hypotheses:

 H1: Transformational leadership has a positive effect on nurses’ adaptive performance.
 H2: Transformational leadership has a positive effect on nurses’ PsyCap.
 H3: PsyCap has a positive effect on nurses’ adaptive performance.
 H4: PsyCap mediates the positive effect of transformational leadership on adaptive performance.
 H5: WPS moderates the relationship between PsyCap and adaptive performance, such that the relationship is stronger when WPS is high.

## 2. Method

### 2.1. Study Setting, Participant, and Procedure

This study employed a quantitative cross‐sectional design to examine registered nurses working in public hospitals across the Gaza Strip, Palestine. The target population comprised nurses delivering primary, secondary, and tertiary healthcare services under the auspices of the Palestinian Ministry of Health. A purposive sampling strategy was implemented between March and April 2023 to recruit participants from the five largest public hospitals, each representing one of the five governorates in the Gaza Strip. To ensure relevance to the study objectives, inclusion criteria required that participants (a) be currently employed as registered nurses, (b) provide direct patient care, (c) have a minimum of 1 year of continuous work experience in their respective hospitals, and (d) be willing to participate voluntarily in the study. Nurses not meeting these criteria or working in administrative roles were excluded.

To determine an adequate sample size, an a priori power analysis was conducted using G∗Power 3.1 [39]. Assuming a medium effect size (*f* ^2^ = 0.15), a significance level of 0.05, statistical power of 0.80, and six predictors in the regression model (transformational leadership, PsyCap, WPS, their interaction term, and two control variables), the minimum required sample size was estimated at approximately 98 participants. Beyond minimum power requirements, prior methodological research indicates that mediation and moderated mediation models benefit from larger samples to ensure stable parameter estimation and accurate standard errors, particularly due to the inclusion of interaction terms and the non‐normal distribution of conditional indirect effects [40, 41]. In addition, recommendations for structural equation modeling suggest that models of comparable complexity with multiple latent constructs ideally include samples of at least 200 cases to achieve reliable and robust estimates [42]. Accordingly, we invited 410 nurses to participate in the study, of whom 334 returned complete and usable questionnaires, yielding a response rate of 81.4%. Hence, the final sample of 334 nurses substantially exceeded the minimum thresholds, providing sufficient statistical power to detect direct, mediating, and moderated effects and supporting the robustness and reliability of the study findings.

Ethical approval was secured from the Directorate General of Human Resource Development and the Palestinian Health Research Council Helsinki Committee, both affiliated with the Ministry of Health. Prior to data collection, formal permissions were obtained from hospital administrations. Following approval from the human resources departments, an online survey was administered using Google Forms. The survey link was distributed through official professional communication channels, whereby assistant supervisors shared the link within existing WhatsApp groups used for routine coordination among nursing staff to ensure that all eligible participants had access. The role of assistant supervisors was limited solely to facilitating the dissemination of the survey link; they were not involved in selecting participants, monitoring responses, or accessing completed questionnaires. Participation was entirely voluntary and anonymous, and neither supervisors nor hospital management had access to individual responses, thereby minimizing potential response pressure or social desirability bias. Participants were able to complete the survey at their convenience using personal computers, tablets, or smartphones. The survey was designed to be concise and user‐friendly, requiring approximately 15–20 min to complete, with mandatory responses for key items to minimize missing data. Two reminder messages were sent at 1‐week intervals to enhance the response rate. Besides, participants were fully informed about the purpose, procedures, and voluntary nature of the study, including their right to withdraw at any point without consequence. Assurances of anonymity and confidentiality were provided, and informed consent was obtained electronically prior to survey submission. The study protocol adhered to the ethical standards outlined in the Declaration of Helsinki [43].

### 2.2. Instrument

We adopted the measurement item scales used in previous studies. The English scales underwent rigorous translation into Arabic, followed by back translation [44]. Two bilingual experts compared the versions to confirm semantic equivalence. The Arabic version’s quality was verified. Additionally, a pre‐test and pilot study were conducted to assess clarity, reliability, and measurement fidelity of the survey before data collection. The pre‐test involved 10 registered nurses from a hospital not included in the main study sample, ensuring that the survey items were clear, unambiguous, and culturally appropriate. Following minor refinements based on their feedback, a pilot study was conducted with 30 registered nurses from another hospital within the Gaza Strip. The pilot data were analyzed to examine internal consistency (Cronbach’s alpha) and preliminary item performance. All scales demonstrated acceptable reliability (Cronbach’s alpha > 0.70), and no items were removed. Minor adjustments were made to improve clarity and ensure ease of completion for the participating nurses. This process ensured that the survey was both methodologically robust and contextually suitable for the target nursing population.

### 2.3. Transformational Leadership

Transformational leadership was assessed using the seven‐item Global Transformational Leadership Scale developed by Carless et al. [15]. The scale is unidimensional and captures employees’ perceptions of transformational behaviors exhibited by their immediate supervisors. Sample items include statements such as “Fosters trust, involvement, and cooperation among team members.” Responses were recorded on a five‐point Likert scale ranging from 1 (never) to 5 (frequently or always). Item scores were averaged to create an overall transformational leadership score, with higher scores indicating stronger perceptions of transformational leadership behaviors and lower scores indicating weaker perceptions. The scale has demonstrated strong psychometric properties in prior research and showed high internal consistency in the present study (Cronbach’s alpha = 0.91).

### 2.4. PsyCap

PsyCap was measured using the 12‐item short‐form Psychological Capital Questionnaire developed by Luthans et al. [24], comprising four subscales: self‐efficacy (e.g., “I feel confident in representing my work area in meetings with management”), hope (e.g., “If I should find myself in a jam at work, I could think of many ways to get out of it”), resilience (e.g., “I usually take stressful things at work in stride”), and optimism (e.g., “I always look on the bright side of things regarding my job”). Participants rated each item on a seven‐point Likert scale ranging from 1 (strongly disagree) to 7 (strongly agree). Item scores were averaged to produce an overall PsyCap score, with higher scores reflecting greater psychological resources and lower scores indicating weaker resources. The scale demonstrated high reliability, achieving Cronbach’s alpha of 0.87.

### 2.5. Adaptive Performance

Adaptive performance was measured using the four‐item adaptivity subscale from Griffin et al. [45], which captures individuals’ capacity to respond effectively to changes in their work roles. Sample items include “I adapt well to changes in my core duties.” Participants rated each item on a seven‐point Likert scale ranging from 1 (strongly disagree) to 7 (strongly agree). Item scores were averaged to produce an overall adaptive performance score, with higher scores reflecting greater adaptability and lower scores indicating less effective adjustment to changing work demands. The scale showed strong internal consistency with Cronbach’s alpha of 0.93.

### 2.6. WPS

WPS was measured using a 10‐item scale adapted from Ashmos and Duchon [33], capturing three core dimensions: meaningful work (e.g., “The work I do is connected to what I think is important in life”), sense of community (e.g., “I feel part of a community in my workplace”), and alignment with organizational values (e.g., “My values are compatible with the values of my organization”). Participants responded on a seven‐point Likert scale ranging from 1 (strongly disagree) to 7 (strongly agree). Item scores were averaged to produce an overall WPS score, with higher values indicating a stronger sense of meaningful work, community, and value alignment, and lower scores reflecting weaker WPS. The scale showed strong internal consistency, achieving Cronbach’s alpha of 0.89.

### 2.7. Demographic Data

Demographic information was collected using a brief, self‐administered questionnaire included in the first section of the online survey. Participants were asked to report their gender, age, educational attainment, work schedule (day or night shift), tenure in their current role, and overall professional experience. These variables were used to describe the study sample and to control for potential confounding effects in the subsequent analyses.

### 2.8. Analysis Strategy

We used SPSS to conduct the descriptive analysis and assess common method variance (CMV). To examine our research assumptions, we applied Partial Least Squares Structural Equation Modeling (PLS‐SEM) using SmartPLS version 4.0. PLS‐SEM is widely recognized for its robustness and predictive capability in modeling complex relationships among latent constructs, and it is particularly suitable for studies in the health and social sciences that employ composite measurement models [46]. In this context, the composites represent the weighted combinations of observed indicators (survey items) used to operationalize each latent construct, capturing the construct comprehensively while accounting for measurement error. Because it is based on composites, PLS‐SEM minimizes the risk of inconsistent or biased estimates [46]. Furthermore, the method allows for the integration of both reflective and formative constructs, facilitating the specification of complex models, including higher‐order structures [47].

Given the reliance on self‐reported measures, potential CMV was rigorously addressed through a combination of procedural and statistical safeguards, in line with the recommendations of Podsakoff et al. [48]. Procedurally, measurement instruments were designed with varying scale endpoints, item wording was carefully calibrated for clarity and conceptual precision, and respondent anonymity was emphasized to reduce evaluation apprehension. Statistically, CMV was evaluated using both Harman’s single‐factor test and the full collinearity assessment approach proposed by Kock [49]. These strategies were implemented prior to hypothesis testing to ensure the validity, reliability, and interpretive integrity of the subsequent analyses.

Following the methodological guidelines of Hair et al. [46], we adopted a two‐stage analytical procedure to ensure the validity of the structural inferences. In the first stage, the measurement model of the reflective constructs was evaluated to establish its psychometric soundness. This stage involved assessing internal consistency reliability, convergent validity, and discriminant validity to confirm that the constructs were measured accurately and distinctly. Establishing a valid and reliable measurement model was a necessary prerequisite for testing the hypothesized relationships at the structural level.

In the second stage, the structural model was evaluated to test the hypothesized direct relationships among the focal constructs, as well as the proposed mediation and moderation mechanisms. Specifically, mediation effects were examined through the estimation of indirect paths using bias‐corrected and accelerated bootstrapping, which provides a robust basis for statistical inference without relying on normality assumptions [50]. In addition, moderation effects were tested by specifying interaction terms within the structural model and evaluating their significance using bootstrapping procedures [46]. This approach allowed for a rigorous examination of whether the strength of relationships varied across conditions.

To enhance internal validity and reduce the likelihood of omitted variable bias, gender and tenure were included as control variables based on theoretical relevance and contextual salience [51]. Gender was controlled due to its significance in the Palestinian context, where sociocultural norms may influence access to resources and role expectations, whereas tenure accounted for accumulated institutional knowledge, familiarity with organizational constraints, and resilience in adversity, which may plausibly affect PsyCap and adaptive performance [52].

## 3. Results

### 3.1. Descriptive Analysis

As reported in Table 1, the sample was predominantly female (*n* = 220, 65.9%), reflecting the gender composition of the nursing workforce. A majority of respondents (*n* = 205, 61.4%) were aged 35 years or younger. In terms of educational qualifications, most participants held a bachelor’s degree (78.7%). With respect to organizational tenure, more than two‐thirds of the respondents (*n* = 232, 69.5%) reported having over 6 years of experience, indicating a relatively experienced workforce. Regarding work schedules, 62.9% of the participants primarily worked day shifts, whereas 124 nurses were assigned to night shifts. Finally, 11.1% of the sample (*n* = 37) reported having less than 2 years of overall professional experience.

**TABLE 1:** Demographic characteristics of participants.

Variables/categories	Frequency (*n*)	Percent (%)	Mean (SD)	Median
Gender			1.65 (0.47)	2
Male	114	34.1		
Female	220	65.9		
Age (years)			2.39 (0.80)	2
Less than 25	32	9.6		
25–35	173	51.8		
35–45	94	28.1		
45 and above	35	10.5		
Educational level			1.99 (0.51)	2
Diploma	40	12		
Bachelor	263	78.7		
Master	25	7.5		
PhD	6	1.8		
Length of experience (years)			3.53 (0.81)	4
Less than 2	14	4.2		
2–4	26	7.8		
4–6	62	18.6		
6 and above	232	69.5		
Work schedule			1.37 (0.48)	1
Day shift	210	62.9		
Night shift	124	37.1		
Professional experience			3.12 (0.76)	3
Less than 2	37	11.1		
2–4	45	13.5		
4–6	90	26.9		
6 and above	162	48.5		
Total	334	100		

*Note: N* = 334 nurses from 5 hospitals.

### 3.2. CMV

To uphold the integrity of the data and ensure the rigor of subsequent analyses, potential CMV and full collinearity were rigorously evaluated. Harman’s single‐factor test confirmed that CMV was not problematic, as the first factor explained only 24.13% of the variance, well below the 50% threshold. In parallel, full collinearity was examined following Kock’s [49] guidelines, with all variance inflation factors (VIFs) reported in Table 2 remaining below the conservative 3.3 benchmark, providing robust evidence against CMV bias. Taken together, these evaluations affirm the methodological soundness of the data and their suitability for reliable measurement and structural analyses.

**TABLE 2:** Results of convergent validity and reliability.

**Construct**		**Outer loading**	**CR**	**AVE**	**VIF**

TL	TL1	0.865	0.913	0.725	2.163
	TL2	0.821			
	TL3	0.829			
	TL4	0.874			
	TL5	0.868			
	TL6	0.839			
	TL7	0.888			

PsyCap	PsyCap1	0.699	0.876	0.500	3.182
	PsyCap2	0.676			
	PsyCap3	0.703			
	PsyCap4	0.702			
	PsyCap5	0.684			
	PsyCap6	0.780			
	PsyCap7	0.732			
	PsyCap8	0.666			
	PsyCap9	0.733			
	PsyCap10	0.655			
	PsyCap11	0.726			
	PsyCap12	0.709			

WPS	WPS1	0.786	0.894	0.594	2.542
	WPS2	0.762			
	WPS3	0.796			
	WPS4	0.765			
	WPS5	0.782			
	WPS6	0.820			
	WPS7	0.765			
	WPS8	0.796			
	WPS9	0.762			
	WPS10	0.758			

AP	AP1	0.924	0.930	0.865	1.658
	AP2	0.939			
	AP3	0.940			
	AP4	0.917			

*Note:* PsyCap—psychological capital, WPS—workplace spirituality.

Abbreviations: AP, adaptive performance; TL, transformational leadership.

### 3.3. Measurement Model

**FIGURE 2:**
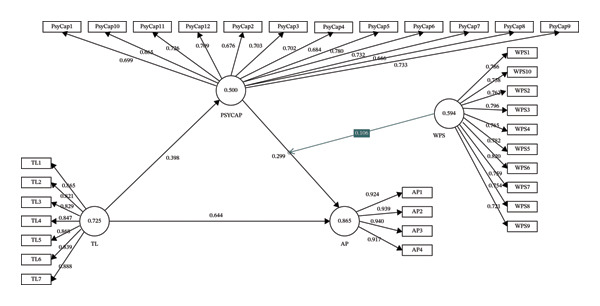
Result of measurement and structural model. Note: TL—transformational leadership, PsyCap‐psychological capital, WPS—workplace spirituality, AP—adaptive performance.

The assessment of the measurement model in PLS followed the guidelines of Hair et al. [46]. Both convergent and discriminant validities were examined. Convergent validity is established when the items of a construct collectively capture the underlying concept. To assess this, we considered composite reliability (CR), outer loadings of indicators, and average variance extracted (AVE). For convergent validity to be satisfactory, indicator loadings should exceed 0.708, since the squared value corresponds to an AVE of 0.50. Loadings between 0.50 and 0.70 may still be retained if the construct’s AVE is at least 0.50. In addition, CR values should meet or exceed the threshold of 0.70. Our results showed that the majority of indicator loadings exceeded 0.708 (Table 2 and Figure 2). The CR values ranged from 0.876 to 0.930, all surpassing the recommended level of 0.70, while the AVE values also exceeded the cutoff of 0.50, thereby confirming convergent validity.

Discriminant validity was further assessed using the heterotrait–monotrait ratio of correlations (HTMT). This criterion evaluates the degree of distinction between constructs, and all HTMT values (Table 3) were below the threshold of 0.90, confirming discriminant validity. To further examine model fit, we used the standardized root mean residual (SRMR), which produced a value of 0.05, below the 0.08 benchmark [47], indicating a good model fit. Overall, based on these criteria, the measurement model demonstrated adequate reliability, convergent validity, and discriminant validity.

**TABLE 3:** Discriminant validity (HTMT).

Variable	AP	PSYCAP	TL	WPS
AP				
PsyCap	0.568			
TL	0.802	0.422		
WPS	0.563	0.707	0.386	

*Note:* PsyCap—psychological capital, WPS—workplace spirituality.

Abbreviations: AP, adaptive performance; TL, transformational leadership.

### 3.4. Structural Model

In line with Hair et al. [46], we assessed multivariate skewness and kurtosis using Mardia’s test. The results indicated significant multivariate skewness (*β* = 1.743, *p* < 0.01) and kurtosis (*β* = 19.575, *p* < 0.01), suggesting a departure from strict multivariate normality. Consistent with methodological recommendations, PLS‐SEM is robust to such non‐normality, allowing reliable estimation of path coefficients and significance levels despite these deviations [46, 47]. The structural model results (Table 4) revealed that transformational leadership exerted a significant positive effect on adaptive performance (*β* = 0.644, *t* = 11.018, *p* < 0.001) and PsyCap (*β* = 0.398, *t* = 6.416, *p* < 0.001). Thus, H1 and H2 were supported. Furthermore, PsyCap demonstrated a significant positive influence on adaptive performance (*β* = 0.299, *t* = 5.134, *p* < 0.001), lending support to H3. Moreover, with regard to the control variables, gender and tenure, both were nonsignificant (Table 4), indicating that the main effects remain robust and are not confounded by these demographic factors.

**TABLE 4:** Hypothesis testing.

Hypothesis	Relationship	Std beta	Std error	*t*‐values	*p* values	BCI LL	BCI UL	*f* ^2^	Decision
H1	TL ‐> AP	0.644	0.05	11.018	0.00	0.507	0.739	0.783	Supported

H2	TL ‐> PsyCap	0.398	0.06	6.416	0.00	0.279	0.521	0.224	Supported

H3	PsyCap ‐> AP	0.299	0.05	5.134	0.00	0.176	0.407	0.185	Supported

H4	TL ‐> PsyCap ‐> AP	0.119	0.03	3.558	0.00	0.062	0.193		Supported

H5	WPS ∗ PsyCap ‐> AP	0.106	0.02	4.602	0.00	0.059	0.150		Supported
	Gender ‐> PsyCap	−0.065	0.09	0.234	0.675	−0.047	0.075	0.004	Insignificant
	Gender ‐> AP	−0.085	0.03	0.867	0.229	−0.015	0.198	0.009	Insignificant
	Tenure ‐> PsyCap	0.060	0.11	0.653	0.246	−0.096	0.255	0.007	Insignificant
	Tenure ‐> AP	0.077	0.08	0.544	0.764	−0.076	0.193	0.002	Insignificant

*Note:* The study applied 95% confidence interval with a bootstrapping of 5000. PsyCap—psychological capital, WPS—workplace spirituality.

Abbreviations: AP, adaptive performance; TL, transformational leadership.

Besides, the effect size (*f* ^2^) was assessed following the benchmarks proposed by Cohen [53], where values of 0.02, 0.15, and 0.35 indicate small, medium, and large effects, respectively. The results revealed that transformational leadership exerted a large effect on adaptive performance (*f* ^2^ = 0.783) and a medium effect on PsyCap (*f* ^2^ = 0.224), as presented in Table 4.

In addition, the explanatory power of the model was further established through *R* ^2^ values, which demonstrated that transformational leadership accounted for 33.8% of the variance in PsyCap. Together, transformational leadership and PsyCap explained 78.3% of the variance in adaptive performance. This high *R* ^2^ reflects the synergistic effect of transformational leadership and PsyCap in sustaining adaptive performance, consistent with COR theory, which posits that interdependent resources accumulate in gain spirals, reinforcing adaptive capacities under stress and scarcity. In resource‐constrained environments such as Gaza, nurses’ adaptive functioning relies disproportionately on the strategic mobilization and interaction of internal and relational resources. Accordingly, the observed explanatory power underscores the centrality of these resource dynamics in predicting sustained adaptive performance, rather than indicating a methodological anomaly. Besides, predictive relevance further was assessed via the blindfolding procedure (*Q* ^2^), yielded values above zero for all endogenous constructs, thereby affirming the predictive validity of the model. Based on Table 4, mediation analysis using bias‐corrected and accelerated bootstrapping confirmed the significance of indirect effects. PsyCap mediated the relationship between transformational leadership and adaptive performance (*β* = 0.119, *t* = 3.558, *p* < 0.01). The corresponding bootstrap confidence intervals excluded zero, validating the mediation effects [50]. These results provided robust support for H4. Finally, moderation analysis tested the role of WPS in the link between PsyCap and adaptive performance. Bootstrapping with 5000 resamples indicated that WPS significantly moderated this relationship (*β* = 0.106, *t* = 4.602, *p* < 0.001) (Table 4; Figure 2). Thus, the proposed moderation hypothesis was supported.

## 4. Discussion

We empirically examined the influence of transformational leadership on nurses’ adaptive performance, identifying PsyCap as the mediating mechanism and WPS as the moderating condition. Consistent with our hypotheses, transformational leadership was positively associated with adaptive performance, lending support to theoretical perspectives that emphasize leadership’s role in fostering adaptability under adverse and demanding circumstances [54]. Within the Gaza healthcare context, we contend that transformational leadership assumes particular importance when environmental demands exceed formal organizational capacities. Nurses operate amid chronic insecurity and acute resource scarcity, where adaptive performance constitutes an ongoing operational requirement rather than a situational response [4]. Under such conditions, adaptive functioning appears linked not only to individual experience but also to leadership‐related support of cognitive, emotional, and motivational processes.

Our findings indicate that transformational leadership is associated with reduced stress and strengthened support structures, which may facilitate adaptive responses under high‐pressure conditions [17]. In acute scenarios, such as mass casualty events, our data suggest that leadership presence is associated with sustained engagement despite cognitive overload or emotional strain [14]. This association underscores the reliance on transformational leadership for maintaining adaptive functioning amid institutional fragility. By framing personal challenges as contributions to collective objectives, transformational leadership appears associated with purposeful effort and reinforced professional meaning [18]. Drawing on the COR theory [19], these relationships can be interpreted as reflecting the capacity of transformational leadership to support resource management processes that help sustain engagement and role functioning under challenging conditions. Our findings further indicate that leaders who restructure workflows, coordinate scarce resources, and provide consistent validation are associated with context‐sensitive decision‐making despite systemic disruption [10]. Observed relationships also suggest that leadership behaviors are linked to reduced anxiety over errors and diminished moral self‐reproach, thereby enabling greater cognitive focus on problem‐solving and attainable objectives while reinforcing collective confidence in sustaining care delivery under demanding conditions [55].

We also reveal a significant positive association between transformational leadership and PsyCap, consistent with prior research [23] and aligned with the COR theory, which conceptualizes leaders as important sources of resource replenishment [19]. In the Gaza context, transformational leadership may function as a critical support system for psychological resources when environmental demands exceed individual coping thresholds. Routine recovery alone may be insufficient to sustain PsyCap, which appears more robust in environments where leadership behaviors actively support the preservation of psychological resources. We observe that leaders are associated with psychosocial environments that facilitate emotional processing and enhance cognitive clarity. Through empowerment, recognition of emotional labor, and affirmation of professional worth, transformational leadership is associated with higher levels of PsyCap dimensions, including hope, resilience, optimism, and self‐efficacy. These capacities appear responsive to contextual demands rather than fixed personal traits. Such practices may restore a sense of agency in settings where nurses confront persistent constraints, embedding resource‐oriented appraisals that sustain engagement [56]. Because recurrent exposure to patient loss, ethical dilemmas, and personal danger accelerates psychological resource depletion, our findings suggest that transformational leadership is associated with redirecting remaining resources toward adaptive reinvestment rather than disengagement [16]. Leaders’ communication and affirmation of professional value also appear related to the integration of challenging experiences into nurses’ professional identity without undermining psychological coherence [57].

We further establish PsyCap as a central driver of adaptive performance, functioning as an internal resource system that enables nurses to navigate volatile clinical environments. PsyCap is associated with sustained motivation, emotional regulation, and behavioral flexibility [27, 58] and appears to operate as a psychological infrastructure supporting engagement and adaptability under prolonged strain. From a COR perspective, PsyCap may function as a resource reservoir that sustains motivation and behavioral recalibration when external supports remain limited [27]. Nurses with higher PsyCap were better able to maintain adaptive functioning despite chronic resource constraints. Adaptive performance in Gaza frequently involves clinical improvisation, resource redistribution, and complex decision‐making under pressure. Our findings indicate that PsyCap is associated with the capacity to navigate these demands without cognitive fragmentation, facilitating rapid reorientation, continuous priority adjustment, and effective decision‐making amid ambiguity. These associations help explain the persistence of adaptive performance under conditions that might otherwise overwhelm individual tolerance. Overall, PsyCap appears linked to the transformation of psychological strain into adaptive action while buffering distress in contexts where systemic dysfunction challenges conventional standards of care [25].

Moreover, PsyCap functions as the principal mechanism through which transformational leadership is partially associated with adaptive performance. This mediating role aligns with the COR theory [19], which conceptualizes leaders as resource passageways facilitating the development, conservation, and strategic utilization of psychological resources. In high‐stress environments such as Gaza, transformational leadership appears related to adaptive performance primarily through its association with nurses’ internal resource configurations rather than solely through observable behaviors. We thus contend that leadership is associated with higher hope through goal articulation, strengthened self‐efficacy through constructive feedback, enhanced resilience through recovery following setbacks, and reinforced optimism through the positive reframing of adversity [59]. Collectively, these associations support the motivation, emotional regulation, and cognitive flexibility that underpin adaptive performance.

Finally, the moderating role of WPS delineates the conditions under which PsyCap is most effectively related to adaptive performance. We observe that WPS strengthens the PsyCap–adaptive performance association, indicating that psychological resources are more effectively mobilized when work is experienced as meaningful and ethically aligned [38]. From a COR perspective, WPS functions as a contextual resource amplifier, enabling nurses to invest PsyCap in adaptive behavior without accelerated depletion. High WPS is associated with alignment between personal values and professional duties [35], thereby sustaining hope, resilience, optimism, and self‐efficacy amid prolonged adversity. In Gaza, where faith, moral obligation, and collective identity are closely intertwined, WPS provides a tangible framework rather than an abstract construct. Nurses draw upon spiritual narratives to interpret suffering, frame caregiving as service to humanity and the divine, and justify personal sacrifice. This orientation appears to protect personal resources, maintain engagement, and enhance PsyCap’s effectiveness in driving adaptive performance [60]. By buffering stress‐induced depletion and reinforcing adaptive motivation under ongoing exposure to trauma and systemic constraint, WPS supports the sustained deployment of PsyCap in dynamic clinical contexts. When work is imbued with spiritual significance, resilience manifests as persistence, optimism sustains effort, and self‐efficacy supports context‐sensitive action even under conditions of personal risk. Through this moral grounding [61], WPS appears to strengthen the conversion of psychological resources into adaptive behaviors that sustain functional capacity and ethically informed clinical decision‐making.

### 4.1. Theoretical Contributions

This study offers several significant and original contributions to the literature on leadership, positive psychology, and performance under crisis, particularly within healthcare systems operating under sustained conflict. First, it is among the first empirical investigation to examine the effect of transformational leadership on adaptive performance within the healthcare sector, filling a substantial theoretical and empirical gap. Prior studies have predominantly focused on how transformational leadership enhances attitudinal outcomes such as job satisfaction or organizational commitment, typically in stable environments. By linking transformational leadership to adaptive performance, this study introduces a new theoretical pathway with implications for healthcare leadership models under extreme conditions.

Second, this research is among the first to test the relationship between PsyCap and adaptive performance in healthcare settings. While PsyCap has been studied in relation to well‐being and attitudinal outcomes, its role in facilitating adaptive performance during prolonged crises remains underexplored. The study extends PsyCap theory by identifying it as a core internal resource that empowers frontline healthcare workers to function adaptively despite extreme workloads and systemic collapse.

Third, the study contributes to management literature by being among the first to empirically test the moderating role of WPS in the PsyCap–adaptive performance relationship. This moderation has not been examined across sectors or theorized within healthcare literature. Introducing this construct as a contextual amplifier expands the COR theory [19] by showing that resource utility is shaped by alignment between internal strengths and moral‐spiritual meaning systems.

Fourth, the study responds to calls for greater contextual diversity in management research [62]. By conducting the study among nurses in Gaza, this study offers a rare empirical contribution from one of the world’s most structurally fragile healthcare environments. While most prior studies draw from Western healthcare systems, this research centers on the lived experiences of nurses operating without consistent access to electricity, supplies, or psychological support. Within this context, WPS functions as a lived existential resource that provides emotional coherence and motivational resilience. Finally, the study contributes methodologically by modeling a moderated mediation mechanism that captures the interplay between leadership, psychological capacity, and spiritual meaning. This structure offers a culturally embedded explanation of behavioral resilience for healthcare workers navigating professional obligations amid trauma and spiritual commitment.

### 4.2. Practical Implications

The findings of this study offer actionable guidance for healthcare systems operating under conditions of chronic instability and resource scarcity, where nurses’ adaptive performance is critical for maintaining service continuity and ensuring quality of care. First, nurse managers should operationalize transformational leadership through behaviors that directly foster adaptability. This entails maintaining visible leadership during periods of heightened demand, providing clear and consistent direction, and modeling calm, ethically coherent decision‐making. Leaders are advised to monitor nurses’ cognitive and emotional capacity, adjust task allocation as necessary, and support less experienced staff through structured guidance. Individualized consideration, timely feedback, and the recognition of autonomous decision‐making further reinforce adaptive responses under conditions of pressure.

Second, leaders should purposefully cultivate nurses’ PsyCap by enhancing confidence, resilience, hope, and optimism. Confidence can be strengthened by empowering nurses to make decisions within clearly defined boundaries and reinforcing their competence through constructive feedback. Resilience should be bolstered through coordinated teamwork and peer support, whereas hope and optimism can be maintained by linking daily responsibilities to meaningful outcomes and acknowledging incremental achievements. These practices directly cultivate the internal psychological resources necessary for sustained adaptive performance. Third, WPS should be strategically leveraged to align nurses’ sense of purpose with their professional roles. Leaders can reinforce this alignment through transparent decision‐making, equitable distribution of workload, and opportunities for reflective practice that connect routine tasks to broader ethical and societal imperatives. Simultaneously, leaders must manage the potential risks associated with strong moral commitment by promoting boundary‐setting and shared responsibility, thereby preventing engagement from resulting in excessive self‐sacrifice or burnout. In sum, the enhancement of adaptive performance necessitates the integrated application of transformational leadership, the deliberate development of PsyCap, and the strategic cultivation of WPS. Healthcare administrators should therefore prioritize leadership development, the fortification of psychological resources, and the establishment of ethically grounded work environments to sustain nurses’ adaptability in high‐pressure settings.

### 4.3. Limitations and Directions for Future Research

Despite offering novel insights into the psychological and contextual factors underpinning adaptive performance, this study has several limitations. First, its cross‐sectional design restricts causal inference. Although theoretically grounded, the lack of temporal separation limits the ability to confirm directionality or rule out reverse causation. Future research should use longitudinal or experimental designs to establish clearer causal links, especially between transformational leadership, PsyCap, and adaptive outcomes. Second, data were collected solely through self‐reported measures. Despite using validated instruments and ensuring confidentiality, reliance on a single source increases the risk of CMV. Incorporating supervisor ratings, peer assessments, or objective indicators of adaptability would strengthen measurement rigor. Third, the study focused exclusively on individual‐level analysis. Important organizational or team‐level influences such as leadership climate or institutional support were not captured. Multilevel modeling in future work could better clarify how contextual variables shape adaptation. Fourth, our study employed the widely validated WPS scale [33]; however, this scale alone may not fully encompass the culturally and contextually specific dimensions of spirituality in Gaza, where faith, community, and existential meaning are deeply intertwined, particularly amid conflict. Future research should adopt mixed‐methods approaches to capture the nuanced experiences of spirituality in this setting and consider adapting or developing measurement instruments that explicitly reflect faith‐based, communal, and existential aspects of WPS.

Fifth, although transformational leadership, PsyCap, and WPS account for a substantial proportion of variance in adaptive performance, nurses’ adaptability in such highly demanding and resource‐constrained settings is likely shaped by a broader constellation of contextual and psychosocial factors that extend beyond the scope of the present model. Accordingly, future research should advance this line of inquiry by incorporating complementary mediating or explanatory mechanisms that more directly capture nurses’ lived and professional experiences, including survival‐oriented coping capacities, family and social support resources, and the psychological sequelae of exposure to acute and cumulative traumatic events. Integrating these dimensions would facilitate a more comprehensive, ecologically grounded, and contextually sensitive understanding of the processes through which leadership fosters adaptive performance under high‐stress conditions. Lastly, findings are based solely on nurses in a conflict‐affected setting, which limits generalizability. Further studies should explore whether the same mechanisms apply across professions and more stable contexts.

## 5. Conclusion

Drawing on the COR theory, this study highlights the role of transformational leadership in enhancing adaptive performance among nurses operating in hospitals marked by chronic uncertainty and acute operational strain. By cultivating PsyCap, transformational leaders mobilize deep personal resources that equip nurses to navigate demanding clinical environments with resilience, confidence, and sustained engagement. The moderating role of WPS further strengthens this effect, fostering a deep sense of moral alignment and professional purpose that energizes constructive responses to adversity. For healthcare systems facing escalating demands and constrained resources, the strategic cultivation of leadership capacity and spiritually meaningful work environments is indispensable. These mechanisms preserve the functional core of hospital operations while advancing nurses’ ability to adapt, innovate, and sustain patient care under conditions that routinely challenge institutional survival. Prioritizing such human‐centered investments is essential for healthcare leaders committed to building psychologically resilient and operationally agile hospitals in the face of enduring complexity.

## Author Contributions

Anas Mahmoud Salem Abukhalifa contributed to the conceptualization, study design, data collection, statistical analysis, manuscript writing, critical revision, editing, and final approval of the manuscript. Mohammed Aboramadan contributed to the conceptualization, study design, statistical analysis, critical revision, and editing of the manuscript.

## Funding

The authors received no specific funding for this work.

## Disclosure

All authors have read and approved the final version of the manuscript.

## Ethics Statement

Ethical approval for this study was obtained from the Directorate General of Human Resource Development and the Palestinian Health Research Council Helsinki Committee (PHRC/HC/112/23), both under the Ministry of Health.

## Conflicts of Interest

The authors declare no conflicts of interest.

## Data Availability

Data are available upon request due to privacy/ethical restrictions.
